# Bioactive and Sensory Di- and Tripeptides Generated during Dry-Curing of Pork Meat

**DOI:** 10.3390/ijms24021574

**Published:** 2023-01-13

**Authors:** Alejandro Heres, Leticia Mora, Fidel Toldrá

**Affiliations:** Instituto de Agroquímica y Tecnología de Alimentos (CSIC), Avenue Agustín Escardino 7, 46980 Paterna, Spain

**Keywords:** tripeptides, dipeptides, proteolysis, dry-cured ham, bioactivity, taste, peptidomics

## Abstract

Dry-cured pork products, such as dry-cured ham, undergo an extensive proteolysis during manufacturing process which determines the organoleptic properties of the final product. As a result of endogenous pork muscle endo- and exopeptidases, many medium- and short-chain peptides are released from muscle proteins. Many of them have been isolated, identified, and characterized, and some peptides have been reported to exert relevant bioactivity with potential benefit for human health. However, little attention has been given to di- and tripeptides, which are far less known, although they have received increasing attention in recent years due to their high potential relevance in terms of bioactivity and role in taste development. This review gathers the current knowledge about di- and tripeptides, regarding their bioactivity and sensory properties and focusing on their generation during long-term processing such as dry-cured pork meats.

## 1. Introduction

A wide range of products such as raw meat, bacon, ham, sausages, and many ready-to-eat charcuterie foods, apart from usable by-products, may be obtained from pork [1]. One of such products is dry-cured ham, which is a popular and high added value product that is consumed worldwide. Its production process varies depending on the country and the traditional manufacturing methodologies employed in the geographic location [2]. However, all of them share the same basic steps in the elaboration protocol: bleeding, salting, post-salting (or resting phase), and drying/ripening, of which salting and drying/ripening are the most crucial steps with influence on the final properties [3,4].

Different factors, such as the methodology and processing conditions, the quality of the raw material, the muscle, type of feed, pork genetics and breed, age at slaughter, and sex, have a strong influence on the biochemical reactions that take place during the production process [5,6,7,8]. Starting from slaughtering and bleeding, metabolism changes are initialized in post-mortem conditions. Enzymatic reactions are predominant, although others of a non-enzymatic nature also occur, and the joint action during the process gives out the typical organoleptic properties of the dry-cured products. Such reactions take place simultaneously to a lesser or greater extent, depending on the stage of processing, but proteolysis and lipolysis are those having the major impact on sensory quality [9].

Since di- and tripeptides are known to impart sensory properties [10], a better knowledge of the proteolytic phenomenon is essential to give an extra value to the product and produce regular batches. During dry-cured ham processing, muscle sarcoplasmic and myofibrillar proteins undergo an intense proteolysis by endogenous muscle peptidases, releasing large amounts of small peptides and free amino acids. The resulting peptides could exert potential bioactivity but are also responsible of organoleptic attributes such as taste [11].

There is solid evidence regarding relatively longer peptides, which are generated along the dry-cured ham elaboration period, reporting their high bioactive potential [12]. However, mass spectrometry techniques have allowed to find them in low amounts in dry-cured ham samples. On the other hand, di- and tripeptides have been far less studied even though they are generated in larger amounts, and therefore they could be more bioactive and exert more relevant sensory properties. Limited research has been performed, majorly due to technical proteomic challenges and trained personnel requirements [13,14]. Indeed, the progressive shortening of larger peptides during the dry-curing of ham suggests the release of a wide variety of di- and tripeptides, which might play an essential role in taste development and bioactivity of the product. It is known that di- and tripeptides are far more bioavailable than larger peptides [15], besides which it has recently been revealed that di- and tripeptides often prove to be crucial for the intrinsic taste of foods, and they can impart taste-modulating properties [10].

This review is focused on the current knowledge about dry-curing of ham as food with a large release of di- and tripeptides during its manufacturing process and also discusses how these di- and tripeptides generated in dry-cured ham contribute to its taste development, as well as about their roles as bioactive agents.

## 2. Short Chain Peptide Generation

The generation of peptides follows similar proteolytic mechanisms between different types of dry-curing processing [7]. Dry aging and salting involves restricting bacterial growth and encourages the growth of beneficial mold. Microbial proteases break down the muscle and connective tissues and penetrate through the meat [16]. As a result, these actions bring about tenderness and taste in the dry aged products [17]. However, microorganisms have no significant role on the global phenomena in the case of dry-cured ham, since low population densities are found inside the samples [18,19]. In contrast, muscle endopeptidases (cathepsins B, D, H, and L, and calpains) and exopeptidases (peptidyl peptidases, peptidases, aminopeptidases, and carboxypeptidases) are the main contributors to the small peptide generation through an extensive proteolysis on myofibrillar and sarcoplasmic proteins during the process [20,21] and under different drying conditions [7]. Indeed, during the last decade, small peptide sequences derived from myofibrillar proteins, such as myosin light chains, actin, titin, troponin T, and sarcoplasmic proteins, for instance glycolytic proteins, myoglobin, and creatine kinase, were identified in dry-cured ham at final time of curing [22].

Dipeptides are first-hand products of the enzymatic action of dipeptidyl peptidases (DPPs) and peptidyl dipeptidases (DDPs) hydrolyzed from the N-terminal and C-terminal of peptide fragments, respectively [12]. Similarly, tripeptides are directly generated by the enzymatic action of tripeptidyl peptidases (TPP) on the N-terminal side of the peptides. Furthermore, free amino acids are also released from the N- and C-terminals through the peptide shortening action of aminopeptidases. Thereby, the joint proteolytic action of the enzymes contributes to a dynamic accumulation of peptides during processing, which has an impact on the development of the typical taste of dry-cured ham [23].

Assessment of the generation of these di- and tripeptides in dry-cured ham requires the adaptation of proteomic strategies to peptidomic scale, which in turn implies facing serious challenges. Such obstacles can begin with the extraction and purification of the small peptides from the samples and separation and isolation techniques prior to MS/MS by using chemical species compatible with the MS analysis [24]. In addition, the complexity of the matrix can lead to signal interferences in the detection as well as the relative low abundance of the di- and tripeptides, and this in joint with their low sequence specificity makes necessary the optimization of the proteomic-based methodologies with the dexterity of trained personnel [13,14,25,26]. It is also important to consider that only a fraction of the peptide extract that reaches the detection yields useful fragmentation ladders, and due to some of these small peptides being present in low concentrations, they can remain masked by the signal of the rest of the peptides. Thus, further development of the peptidomic approaches is needed to make the identification and quantitation of the di- and tripeptides more practical [27].

## 3. Taste Perception

Gustatory papillae are folded structures of the epithelial tissue present in tongue and other parts of the oral cavity which contain specialized taste buds, the basal lamina, and the proper lamina. The latter is where nerves irrigate. Taste buds are apical structures that house taste receptor cells [28], presenting microvilli in the apical area and extending to the basal lamina, where they establish synaptic connections with nerve fibers. These connections allow to fulfil epithelial, neuronal, and secretory functions [29], integrating interoceptive (hunger, satiety, specialized appetites) and exteroceptive (vision, olfaction, somatosensation) signals and generating behavioral responses to taste stimuli [28].

Taste receptors are oriented to the oral cavity, allowing extra-organism and extracellular appreciation of nutrients (usually water-soluble chemicals) to select suitable food sources. The classification of receptors in humans can be carried out basing on their specialization to perceive the following tastes: sweet, salty, bitter, sour/acidic, and umami [30]; the latter is an Asian term that refers to the taste of monosodium glutamate (E621), commonly used as a taste enhancer. Structurally, taste receptors belong to the G protein-coupled receptors (GPCR), but also can be ionic channels [31]. In this respect, GPCR-related type 1 taste receptors (T1R) and type 2 taste receptors (T2R) are expressed in different subsets of taste receptor cells [28]. T1R receptors are known to form heterodimers, whereas T2R receptors generally act as monomers. The best-characterized receptors in detecting amino acidic sources are those formed by heterodimerization of T1R1 and T1R3 receptors. Taste receptors are also expressed in other tissues involved in food recognition and digestion. For example, neutrophils express T1R1/T1R3 heterodimers and are stimulated by A and S amino acids [30].

T1R1/T1R3 allow to perceive umami compounds such as E and other L-amino acids, but other receptors are also involved in the detection of umami compounds: metabotropic glutamate receptors mGluR4 and mGluR1. Interestingly, 5′ nucleotides exert a synergistic augmentation of umami taste when present in small amounts alongside E [31]. In this regard, guanine concentration, nucleotide concentration, and their combination, which enhances the umami-related characteristics, are increased up to 540 days of ripening in dry-cured ham [32]. Notwithstanding, in addition to the T1R1/T1R3 heterodimer, another receptor or possibly a receptor complex is involved in the detection of L-amino acids and inosine [33]. This is the case of calcium-sensing receptor (CaSR), another GPCR involved in perception of umami taste [34].

T1R2/T1R3 heterodimer participates in sweet perception and detects D-amino acids. Sugars and dipeptide sweeteners bind to the N-terminus as well as sweet proteins, which also interact with C-rich domains of the heterodimer. Instead, T2Rs exhibit functional polymorphisms that result in a broad and overlapping range of ligand sensitivities implying that this family responds to an enormous range of bitter-tasting chemicals. Sour taste stimulus is less known, is proposed to be transduced by membrane depolarization and proton and ion membrane channels, and is directly correlated with concentration of organic and mineral acids [31].

## 4. Taste Evaluation Assessments

The analysis of emotional responses from panelists when consuming Iberian dry-cured ham evidenced a correlation between “intense”, “authentic”, and “pleasant” and the intramuscular fat content. In contrast, for the case of Serrano and Curado dry-cured hams, saltiness seemed to be linked to “ordinary”, “indifferent”, and “dissatisfied” attributes. On the other hand, positive emotions such as “desirable”, “pleasant”, and “authentic” elevate the overall liking scores [35].

Sensory assessments are laborious processes which imply the availability of trained panelists to assess their basic capacities of perception, their training and finally reach an adequate qualification, besides which it is important to consider their demographic and socio-economical characteristics apart from their consumption trends [36], as taste preference has a genetic and cultural background [37]. In addition, statistical methods and strict control of the experiment design are required for the development of the analysis [38].

In contrast, in silico and computational predictive methods are gaining attention as possible combining techniques for their availability and smartness. In this context, an artificial neural network model was developed for the prediction of the sensory parameters of dry-cured hams of 24 months of elaboration based on near-infrared spectroscopy information. A relationship was established between sensory dry-cured ham attributes and different parameters for the perception of each attribute, allowing to build a neural network from which it was possible to obtain predictions of the sensory attributes with satisfactory correlation. Aftertaste, rancidity, and flavor intensity showed the highest correlation in relation to odor and taste in dry-cured ham [39].

In addition to the analysis of sensorial characteristics of dry-cured ham through different approaches, active efforts have been made for the identification of the compounds responsible for the attributes, besides which there is a growing interest in the elucidation of the sensing mechanisms and taste transduction. An interesting study evaluated the taste of dry-cured ham peptide extracts-derived gel filtration fractions and identified some dipeptides [40]. Since di- and tripeptides result from the combination of two and three residues, respectively, there are 20 × 20 = 400 and 20 × 20 × 20 = 8000 possible dipeptides and tripeptides, without considering muscle proteins sequences. The sensory assessment of all of them would be expensive, tough, and time-consuming, as it would require them to be synthetized at enough amounts, with high purity, and also to count with a trained sensory panel. Thus, in silico approaches can suppose a convenient methodology to address these issues.

Basing on residue physicochemical properties, such as molecular weight, bulkiness, polarity, etc., computational analysis can be performed to build models establishing quantitative and qualitative relationships between the peptide composition and their impacting taste. This is the case for multiple linear regression, PCAs, PLS, and QSAR analysis developed to predict the bitterness of di- and tripeptides [41,42,43]. Intriguingly, machine learning-based methods recently started being developed for peptide taste prediction [44,45].

Taste-active peptides are those which can interact to TLRs triggering a nervous response. Bottleneck issues exist in determining these mechanisms due to difficulties in the study of the TLRs, such as recombinant expression and protein purification [46]. In this context, salty and sour perception involves the detection of ions which triggers the activation of a voltage-gated calcium influx to initiate neurotransmitter release from a conventional chemical synapse. However, bitter, sweet, and umami stimuli activate GPCRs that initiate a common signaling pathway involving calcium release from stores via activation of inositol triphosphate receptors and leading to the activation of the gustatory nerve [47]. Functional heterologous expression in calcium imaging assays comprises a set of sensitive ex vivo techniques to approximate the activities of taste-active compounds. For instance, HEK293T cells expressing the bitter receptor have been used for the evaluation of bitter compounds [48,49]. Moreover, complementary analyses involve the study of mRNA expression of the corresponding taste receptor genes in joint with immunocytochemical localization as insights of taste-enhancing activity [50].

Unfortunately, ex vivo experiments cannot fully recreate the native cellular microenvironment. To solve this limitation, a novel image-based screening platform that enables high-throughput functional screening of taste cells in vivo was developed. By infusion through a microfluidic channel, it is possible to control the delivery of multiple liquid-phase tastants on a stabilized tongue, and the stimuli is monitored by performing serial two-photon imaging on the taste bud at ~6 Hz. Calcium imaging in vivo assays have also been carried out for monitoring of the geniculate ganglion of a live, anesthetized laboratory mouse to measure the responses of ensembles of these neurons to taste stimuli [51]. In addition, another strategy consisted of immobilizing the human umami receptors (T1R1, mGluR1, and mGluR4) onto a precoated glassy carbon electrode to mimic the cascade amplification system [52].

The applications of mutation testing, mathematical modelling, and molecular docking can enhance knowledge about molecular mechanisms of the receptors [46]. Receptor and ligand structures are minimum requisites for computational molecular docking and interaction modelling. These structures might be determined experimentally or by computer-based prediction methodologies. As an example, molecular docking methodologies have allowed to determine the main human mGluR peptide-interacting residues, which are Trp110, Gly163, Ser164, Ser165, Ser186, Tyr236, Asp318, Asp319, Ala329, and Gly379 [53,54].

Currently, the crystallographic structures of some human taste receptors are still unresolved; such is the case of T1R1/T1R3. Precisely, a new active conformation for the human umami receptor mGluR1 has lately been determined (PDB ID 7DGE) [54]. Fortunately, homology modelling is employed to generate a 3D structure model of an unknown receptor and to understand its molecular mechanisms. This approximation depends on the theoretical relationship expected between the three-dimensional structures of proteins that have evolved from a common ancestor protein while retaining substantial similarity in both amino acid sequence and overall function [55].

This strategy has been used to study T1R1/T1R3 ligands with umami taste. The active sites located in T1R1 and T1R3 correspond, in a similar manner, to the E-interacting sites in mGluR1. Therefore, it is feasible to build reliable homologue models of T1R1/T1R3 [46], but also from other TLRs. Studies following this strategy determined T1R1/T1R3 (umami receptor) active residues were Gln52, His71, Tyr107, Asp147, Ser148, Thr149, Arg151, Ala170, Ser172, Asp192, Tyr220, Arg277, Glu301, Gln302, Ser306, Gly304, His308, His364, and Glu429 [56,57,58]. Following this theoretical focus, glutamate receptors’ crystal structures have served to homology model T1R2/T1R3 (sweetness receptor), whose active residues were predicted to be Ala49, Ser53, Ser59, Lys60, Glu148, Asp169, Arg172, Lys174, Asp188, Asp216, Leu245, Pro246, Ser276, Leu313, Tyr314, Asp456, and Ser458 [59,60]. In particular, dry-cured ham-derived peptides KGDESLLA, SEE, ES, and DES were calculated to be recognized by Ser146 and Glu277 in T1R3 [61].

For the case of the bitter receptors belonging to the T2R family, residues Phe88, Met89, and Tyr242 were predicted to be maintained in various dockings. However, more residues involved in the recognition of bitter peptides might interact [62,63].

To conclude this section, it is sensible to comment the high importance of the availability of large combinatorial libraries and peptides databases that allow an efficient implementation of high-throughput screening of taste-active compounds. Special attention must be paid to BIOPEP-UWM database, which consists of a set of useful in silico proteomics tools and also contains a wide database of bioactive and sensory peptides [64,65].

## 5. Taste Development during Dry Curing

Sensory analysis is an essential part of assessing the quality of foods. Color, intensity of odor and taste, saltiness, and fat content have been pointed out as the main descriptors to represent the sensory attributes of dry-cured ham. Dry-cured hams of 6 months of elaboration tasted bitter, piquant, and metallic. While some of these sensory attributes were ameliorated with the progression of the dry-curing process, others such as sweetness, “matured”, and “aged” flavors intensified in samples of 18 months of elaboration [36]

The objective of dry-curing is not only to increase the life of the product, guaranteeing its healthiness, but to obtain nutritional and sensorial properties which are attractive to the consumer [4]. The most frequent perceptible characteristics or organoleptic properties, termed as “attributes”, can be categorized in physical aspect, smell, rheological aspect, and gustative perception [38,66]. There have been studies regarding the influence of different manufacturing parameters on the final organoleptic characteristics of the product, and taste might be the most sensitive to protocol changes. For example, taste intensity can be increased by modernizing the production of Jinhua ham, and this improvement in taste was associated with a higher degree of proteolysis [67].

Among the organoleptic properties, taste is considered as one of the most important factors determining attractiveness of food products and consumer preferences. The attributes encompassed in this category are cured ham and rancid flavors, saltiness, pungency, sweetness, and acidity [68]. Amino acids and small peptides are key contributors for the taste perception in dry-cured hams [69]. Despite all this, less is known about the influence of di- and tripeptides.

Taste-active peptides, amino acids, and amino acid derivatives play a crucial role in the taste formation of dry-cured hams and sausages, but other taste contributors generated during ripening are 5′-inosine monophosphate (IMP) and guanine, enhancers of umami taste. During long ripening periods, IMP is converted to hypoxanthine and inosine, thereby increasing the intensity of bitterness [32]. The level of lipid oxidation increases with the aging time, thus resulting in the release of compounds that react with the protein degradation products and impart an intense flavor [70].

## 6. Taste of Amino Acids

L-amino acid detection mechanisms are particularly important as they could be targets for altering taste properties of food, making it more or less desirable [33]. The most frequent tastes between amino acids are bitterness, umami, sweetness, and sourness [32,69]. Their main taste attributes are shown in Table 1.

The generation of free amino acids in dry-cured meat products is significantly high and their concentration, several hundreds of mg/100 g, generally exceeds the taste threshold, strongly influencing the taste of dry-cured products [69]. A monotonic increase was observed during ripening in dry-cured ham of each amino acid, except for Q and C. N, L, and I presented the highest accumulating rates, and it is attributed to proteolysis [32]. Amino acid profiles differ between ham types. Since not only the total concentration of amino acids but also the balance among them contributes to taste, taste perception varies depending on dry-cured ham. For example, K, Y, D, A, and E are the most abundant free amino acids in the ripening of Iberian ham and strongly influence the taste of dry cured products [69]. However, intense proteolysis of sarcoplasmic and myofibrillar proteins could result in the occurrence of unpleasant texture and taste such as intense bitterness and adhesiveness due to the accumulation of unpleasant-tasting peptides and free amino acids as it occurs in defective hams [73].

## 7. Taste of Di- and Tripeptides

The main contributors to the development of the typic dry-cured ham taste are the non-volatile compounds amino acids and small peptides as a result of the intense proteolysis [74]. Most frequent peptide-exerting tastes are umami, sweetness, and bitterness [43]. There is limited data on the identification of peptide sequences that directly contribute to the taste of dry-cured meats, despite the fact that knowledge about sensory peptides provides a useful tool to produce meat-derived products with desirable taste and bioactivity qualities.

Bioinformatic analysis has become a relevant tool for research on meat proteins as a source of taste-active peptides and amino acids prior to in vitro and in vivo protocols, allowing to confirm mostly di- and tripeptides are the main responsible compounds for taste sensation. In this sense, an in silico study evaluated porcine sarcoplasmic and myofibrillar protein sequences as potential precursors of taste-active peptides under processing conditions, and it was demonstrated that the number of peptides with sensory activity is proportional to the protein fragment length. However, the release of peptides depends on the enzyme specificity. The results showed that myofibrillar proteins are a more abundant source of taste-active components of meat than sarcoplasmic proteins, and particularly, myosin-2 protein. Nevertheless, the most abundant source of amino acids and taste-active peptides is troponin C from skeletal muscle, contributing to the perception of all five basic sensations [75].

Extensive hydrolysis during dry-curing processes, with the consequent release of small peptides and amino acids, has been reported in several studies [7,22,76,77,78,79,80]. Many of the resulting compounds determine the taste by themselves and by transformation due to other biochemical pathways involved.

Table 2 compiles the taste of di- and tripeptides identified to date in dry-cured ham.

Remarkably, a specific amino acid taste is not always encoded in the structure of a peptide; for example, the amino acid E results in mouthfeel perception of a sour and umami taste, while as a dipeptide EE allows the taste to be bitter and salty [75]. Some peptides and other taste-active components, in appropriate concentrations, could mutually strengthen their tastes. Such peptides might have little or no taste, and more than half contain the acidic amino acids E or D [94].

### 7.1. Bitterness

Evidence suggests that aging rises bitter small peptides. However, the role of bitter peptides may be counteracted by the combination with other taste-active compounds in adequate proportion, otherwise, an unbalanced accumulation could generate an unpleasant bitter taste [81]. Since excessive bitterness, softness, and adhesiveness are the main sensory and textural defects in products such as dry-cured ham [80], the understanding about biochemical taste development would allow to control the formation of unpleasant characteristics.

Multiple linear regression models have been used to determine that hydrophobicity and the presence of branched side residues or ring in a di- or tripeptide sequence, as L, I, V, Y, F, influences their bitterness, specifically, the presence of a bulky C-terminal amino acid in a peptide sequence [43]. The hydrophobic group of an amino acid is involved in the mechanism of binding to the bitter taste receptor [45].

F and Y residues have been described as the main amino acids affecting bitterness in a sequence [75] and peptides FFF, FFG, FG, FGF, FGG, GF, GFF, GFG, GGF, GGY, GY, GYG, GYY, YG, YGG, YGY, YY, YYG, YYY have been corroborated as bitter tastants—concretely, when F residue is located at the C-terminal position [95,96].

Due to its imino ring, P also plays a determinant role due to the fact that it causes a folded structure within the peptides, favoring conformational changes which allow to bind to the bitter taste receptor. Despite the fact that PG has been sensed as flat, di- and tripeptides formed by P residues, and in combination with G or hydrophobic residues such as F or Y, have been described as bitter. However, bitterness weakens or disappears when P residue is located at the N-terminus. On the contrary, basic amino acids such as R located at the N-terminus have been suggested to favor bitterness, probably due to the imino group from P interacting with the binding unit of the receptor whereas the guanidino group from R residue interacts with the stimulating unit. FPF, GRP, KPF, PFP, RPG, and YPF are examples of potent bitter sequences. Additionally, P and R homopeptides have demonstrated that bitterness is not boosted with the peptide length [43,97]. Despite the fact that the dipeptide RP has been found to possess strong bitter taste, its reversed amino acid sequence has a weaker bitter taste, and with the replacement of the R residue with other basic amino acids such as K, it was found that the bitterness is kept. The observations indicate that basic residues located at the C-terminal position improve bitterness [96].

All the same, L, I, and V are considered as extremely bitter with unpleasant taste and odor [43]. Bitterness is incremented with L frequency in the peptide, as demonstrate LL and LLL homopeptides. Peptides GGL, GL, GLG, GLL, LG, LGG, LGL, and LLG also are bitter, with the particularity that a C-terminal L residue in tripeptides pronounces bitterness [98].

Experimental designs have reported the side chain skeleton of the residue within a peptide should consist of at least three carbons for exhibiting bitter taste, while peptides with residues with less than three carbons are not bitter. In agreement with these conclusions, hydrophobic residues in cooperation with the presence of V residues, promotes bitter taste as it is shown in bitter peptides FIV, FV, GGV, GV, GVV, IV, LV, VD, VE, VF, VI, VIF, VL, VVV. Additionally, attending to differences between L- and I-containing peptides, the linear side chain seems to exert a more intense bitterness than does the branched chain [99].

Partial least squares regression analysis showed the intense degradation of myosin and troponin in dry-cured ham has a key role in bitterness [67]. Dipeptides containing hydrophobic amino acids such as IV, LE, ID, and PL have been reported to impart a bitter taste in Serrano dry-cured ham, as do their respective isolated forms [40]. Bitter dipeptides GF and LL have been estimated to be generated in Parma dry-cured hams [81] and bitter sequences AD, DL, EA, EE, EF, EI, EL, GP, IF, IL, KP, LA, LG, LL, PA, PK, PL, PP, RG, VE, VF, VY are probably generated in dry-cured ham [75].

In addition, various bitter dipeptides have been quantified in dry-cured hams. The dipeptide PA was quantitated in Spanish dry-cured hams and the dipeptides PG and VG were detected in Norwegian dry-cured hams [82]. The same was stated for the dipeptide PL [83]. Moreover, the dipeptide GL was also determined to be increased in concentration in Prosciutto-like processed dry-cured hams [32]. PA and VG have also been quantitated in Spanish dry-cured hams with a traditional processing and under a low-salted elaboration condition [84,100].

### 7.2. Umami

Umami sensation is used to describe the taste of savory and meat broth foods. Umami molecules contribute to the perception of savory taste and increase other taste intensities [101]. For this reason, taste evaluation of umami peptides is impacted by other peptides, nucleotides, and cations, and possibly by the dissolved media.

The frequency of E and D amino acid within the sequence is a key factor establishing salty and umami dipeptides. Combinations of both amino acids and with other residues along the sequence impart umami and brothy tastes. For example, EE, ED ES, and EGS have similar umami tastes but weaker than that of monosodium E. By contrast, GE and AEA are stronger than monosodium glutamate [101]. In addition, ED, EL, ECA, EGS, and DES have also been qualified as umami tastants [102].

E residue containing di- and tripeptides remarkably increase during the extended aging of ham, thus acting as permanent taste-active compounds [103]. In concordance with E-rich oligopeptides predicted by Kęska and Stadnik [75], dipeptides DE, EA, EE, EK, EL, VE, probably generated in dry-cured ham, impart umami taste as well as bitterness or sourness [86].

Some perceptions of taste are in controversy due to the peptides imparting different taste when they are synthesized compared to that sensed when isolated from the food source. Such discrepancies could be attributed to the preparation method, the isomeric structural differences, and the variation in the spatial structure between peptides from hydrolysates and synthetized ones. AE, DA, DE, DL, EK, ES, EV, and KG are several examples [101].

Related to these observations, PE could act as an enhancer of umami taste in dry-cured products [86]. Moreover, short peptides found in Jinhua ham, VE, PE, PAQ, and NGG showed umami taste, as well as hydrophobic and alkaline amino acid-consisting peptides AH, HP, VY, and LH, which might impart umami taste [104]. In the same line, E-rich dipeptides EE, EF, EK, and DA were suggested to be released from myosin light chain isoforms in dry-cured ham [73]. Gastrointestinal digestion simulation of peptides from Jinhua ham demonstrated that Jinhua ham is a source of umami peptides such as EL, EV, RL, EEL, and ESV [73]. Several umami-imparting dipeptides concentrations have recently been found to be increased with the progress of the elaboration, such as DA, DG, EE, ES, and EV from Spanish dry-cured hams, except for VG [84,87,100]. The dipeptide AH was discovered in Jinhua dry-cured hams [83], and it was also identified in Spanish dry-cured ham [87]. Additionally, EE was determined to be increased in Prosciutto-like processed dry-cured hams [32]. In addition, the umami peptide DK can probably be released during dry-curing [22,58]. Even umami dipeptides GS, KP, PN, and SY have been identified in hydrolysates from porcine bone protein extracts [105]. Finally, dry-cured ham-occurring dipeptides AH, DA, DG, EE, ES, EV, and VG have also been evaluated in in silico molecular docking against mGluR1, reporting glutamate-like interactions, but the data also revealed that other residues from the receptor might be involved in the docking with the ligands [87,106].

In a similar activity to E amino acid, pyroEP-Z (Z = any residue) tripeptides impart umami taste. These peptides are generated during heating by cyclization of corresponding α-glutamyl- or α-glutaminyl dipeptides but also species used in fermentations as *Lactobacillus helveticus*, *L. delbrueckii* subsp. *bulgaricus*, and *Streptococcus thermophilus* were reported to have pGlu cyclase activity [72].

### 7.3. Sweetness

Sensory evaluating assays reported short peptides such as AA, AAA, AAG, AGA, AGG, GAA, GAG, and GGA impart sweet taste and some of them at a threshold compared to that of sucrose [99].

**Figure 1:**
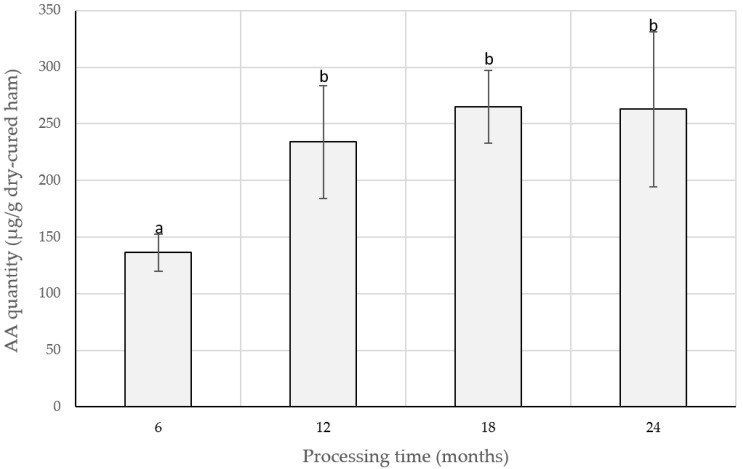
Evolution of AA concentration (µg/g Spanish dry-cured ham) during 6, 12, 18, and 24 months of processing. Different letters indicate significant differences among the month values at *p* < 0.05. Reproduced from [90] with permission.

Short sequences AA, AAA, AGA, AGG, EV, GAG, and GGA have been identified within porcine muscle proteins and have been correlated with sweet taste [75]. AA and GAG also have been found to be probably generated in dry-cured ham [88]. In fact, the dipeptide AA was found in Spanish and Prosciutto-like processed dry-cured hams, and its concentration was suggested to increase during the elaboration, as shown in Figure 1 [32,90].

### 7.4. Sourness

D- and E-containing peptides such as VD and VE have been qualified as sour-tasting agents and with slight bitterness, or a mixed taste, whereas a merely sour taste is perceptible in DV or EV linking the C-terminal V [99]. Despite the fact that L residue confers bitterness and remarkably in C-terminal position, when it is bound to an acidic amino acid in a dipeptide, only a sour taste is retained while bitterness disappears [98].

AED, DD, DE, DV, DEE, DES, ED, EV, VD, and VE peptide sequences detected in porcine muscle proteins have been targeted to impart sour taste (Paulina Kęska and Stadnik, 2017), and sour-tasting dipeptides VE and GE have been found in dry-cured ham. This reveals the frequency of D residue in short peptides, which could be linked to sour perception, agreeing with the hypothesis consisting of peptides present in dry-cured ham as DV could impart sour taste [40], as well as DE, also probably generated during dry-curing [88].

### 7.5. Saltiness

NaCl masks metallic and bitter tastes and enhances umami taste. Amino acids and peptides may enhance the salty taste, as it is known the salty taste of dry cured meat is correlated to the concentrations of E and D [72]. Salt is a key element of dry-cured products, but also it inhibits enzymatic reactions [107] and thus, water activity and NaCl concentration within the product play an important role in sensory peptide releasing and taste development.

Among all saltiness proposed imparting sequences [75], DE is probably generated in dry-cured ham [22,88], and the salty dipeptide AR was found Jinhua dry-cured ham [83].

### 7.6. Kokumi Activity

Kokumi compounds are non-taste active substances, having only minimal aroma in water [108], but they enhance the taste intensity of other sapid substances by modulation of the signal transduction from the taste receptors to the brain. Thus, kokumi activity can be considered a type of taste interaction between taste-active and non-taste active compounds. In the presence of sensory compounds within the food matrix, such species can cause tastes that cannot be explained by the five basic tastes alone, such as thickness, continuity, and mouthfulness. More precisely, it has been demonstrated that kokumi peptides act as agonists of the CaSR [109], binding to the large extracellular Venus flytrap domain of the receptor through their α-amino and α-carboxyl groups [108,110].

γ-Glutamyl peptides are distinctive kokumi agents. Microbial enzymes γ-glutamyl transferase, γ-glutamyl transpeptidase, or γ-EC synthetase are involved in their production during fermentations [72]. Additionally, endogenous enzymes from the glutathione cycle catalyze these reactions [111]. Particularly, γ-glutamyl dipeptides are mainly generated in foods by γ-glutamyl transpeptidases or γ-glutamyl transferases from amino acids [72]

γ-Glutamyl di- and tripeptides, glutathione (γ-ECG), as well as γ-EVG, γ-EA, γ-E-Abu-G (Abu: α-aminobutyric acid), γ-EC, γ-EE, γ-EG, γ-EH, γ-EL, γ-EM, γ-EQ, and γ-EV have been described as kokumi tastants [94,108,109,112,113]. Interestingly, while α-EF is qualified as bitter and sour, γ-EF is not bitter, but sour. Thus, treatments with γ-glutamyl transpeptidases could be a way of enzymatic debittering of foods for taste improvement [114]. In addition, γ-glutamyl di- and tripeptides that comprise sulfur-containing amino acids can constitute important upstream precursors of volatile flavory compounds and act as flavor-modifying agents, but there is little information available. When sulfur-containing peptides are in presence of umami compounds, they show common intrinsic taste depending on the functional group of the sulfur-containing amino acid; for example, glutathione and γ-EC comprise a common sulfhydryl-methyl side residue and present similar intrinsic taste. This could be explained by the fact that the presence of a medium-sized, aliphatic, neutral, and non-polar substituent at the second amino acid residue is preferable to bind to the CaSR. However, γ-EC and other evaluated γ-glutamyl peptides such as γ-EM exert lower continuity ratings than glutathione, regardless of the structure of the substituent on the sulfur atom, the oxidation state of the sulfur atom, or the peptide length [108].

γ-EI, γ-EL, γ-EF, and γ-EY have been detected in Parma dry-cured ham showing a remarkable increase during ham extended aging time, being unsuitable for further enzymatic breakdown and acting like permanent taste-active compounds. Available glutamyl substrates, activity of γ-glutamyl transpeptidase, and free amino acids are key factors for their generation. Thus, the strong link between the proteolysis degree and the aging time may turn these dipeptides into effective markers of long-aged dry-cured meat products [81,92]. More recently, γ-EF, γ-EW, and γ-EY have also been reported in Prosciutto dry-cured hams [93]. These results, in joint with the fact that the enzyme γ-glutamyl-transferase has also been found in different tissues of the pig [115], suggest that this enzymatic activity might occur during the processing of dry-cured ham, contributing to the development of the typical taste.

## 8. Bioactivity of Di- and Tripeptides

The possibility of preventive strategies or contributing beneficially to therapeutic treatments through the ingesta of bioactive peptides is of high interest in research to reduce incidence of risk diseases and healthcare costs. Protein-rich foods have a great nutritional relevance as providers of essential amino acids, but also as sources of bioactive peptides released by enzymatic actions during their manufacture process, reactor-controlled hydrolysis or digestion and postprandial metabolism. Bioactive peptides are generally 2–20 amino acids in length and are capable of exerting positive health effects in the human body such as antihypertensive, antioxidant, anti-inflammatory, and antidiabetic activities, among others [116]. Since peptides can present low toxicity and accumulation in tissue [117], dietary bioactive peptides could mean a simple way of preventing infection and diseases, avoiding treatments with synthetic peptides and drugs accompanied by their side effects. Additionally, the economic impact on healthcare in future years because of bad habits and ageing of the population might decrease with the development and use of bioactive peptides. The diverse function of peptides is indivisible with amino acid composition, terminal amino acid, chain length, total molecular weight, hydrophobic property, and spatial structure [116].

Endogenous muscle proteins from meat products can suppose a great source of bioactive peptides through proteolysis action, as occurs in fermentations and curing processes [118]. For example, an in silico study reported potential bioactive short peptides predicted to be released from porcine myofibrillar proteins by hydrolysis with pepsin, trypsin, and chymotrypsin [75].

Numerous empirical studies have reported the generation of potential bioactive peptides in dry-cured ham, and it is remarkably a clearly beneficial effect against cardiovascular diseases and metabolic disorders. It has been proved that peptides generated from endogenous muscle proteins during dry-curing of ham exert in vitro angiotensin I-converting enzyme (ACE-I) inhibitory activity, antioxidant activity, dipeptidyl peptidase IV (DPP-IV), α-glucosidase inhibitory activity, antioxidant, anti-hyperglycemic, anti-inflammatory, and antilisterial activities [91,119,120,121,122]. In fact, some in vivo and interventional studies have also been conducted, demonstrating antihypertensive and anti-inflammatory potential [123,124,125,126,127].

Peptidomics allows to find bioactive motifs within the sequence of peptides basing on the composition, structure, and post-translational modifications of the residues that are present. The outcomes from this analysis, typical from in silico-based research projects, serve to test new bioactive peptides. ACE-I inhibition and antioxidant capacities have been largely studied, where there is a wide gap about the peptide sequences identified in other less known bioactivities [116,128]. Furthermore, food-derived di- and tripeptide’s bioactivities remain less far examined [129].

Despite many in vitro and in vivo studies demonstrating the bioactive potential of peptides, different approaches are needed in terms of bioavailability, since some peptides may be susceptible to partial or total loss of activity as a result of food matrix interactions and further hydrolysis by digestive enzymes and intestinal microbiota. Finally, peptides must reach their target sites in an active form and in significant quantity to exert their beneficial effects [12]. Di- and tripeptides are absorbed more efficiently than larger ones, which are prone to be hydrolyzed by enterocyte peptidases [15,130]. It has been reported that peptides having low molecular weights are less prone to proteolytic digestion and are easily absorbed in the intestine as they can easily cross the intestinal barrier and thus exhibited more potency during in vivo studies [131]. In general terms, three minimum structural features are required for peptides to be transported through the intestine barrier: a free C-terminal carboxyl group, preferably in L-configuration, or an amino group or a weak basic group at the N-terminus, an overall charge of less than two positive units [132]. This knowledge would constitute a starting point for the development of peptide-based therapeutic treatments.

Table 3 congregates bioactive di- and tripeptides identified to date in dry-cured ham.

**Table 3:** Dry-cured ham (DCH)-derived di- and tripeptides’ bioactivities.

Sequence ^a^	Origin (Curing Months)	Reference
***Antihypertensive activity ^b^***		
AA, GP, KA, RP, and VY	Spanish DCH (9 m)	[88,91]
AA	Traditional and low-salted Spanish DCH (6, 12, 18, and 24 m)	[22]
AKK, PAP, SGP, and TNP	Spanish DCH (2, 3.5, 5, 6.5, and 9 m)	[88,133,134]
LGL, ALM	Parma DCH (18 and 24 m)	[135]
EW, IF, GA, PL, and VF	Iberian DCH (24 m)	[122]
GA and VF	Traditional and low-salted Spanish DCH (6, 12, 18, and 24 m)	[87,100]
EL, EV, RL, EEL, and ESV	Jinhua DCH (6 m)	[15]
LR, NR, and EF	Spanish DCH (9 m)	[88,136]
YA	Laowo DCH (12, 24, and 36 m)	[137,138]
AW	Spanish DCH (6, 12, 18, and 24 m)	[90]
***Antioxidant activity***		
AY, EL, KP, VY, and EAK	Spanish DCH (2, 3.5, 5, 6.5, and 9 m)	[88,133]
AW	Spanish DCH (6, 12, 18, and 24 m)	[90]
Anserine and carnosine	Spanish DCH (10 m)	[120,139]
***Antidiabetic activity***		
KA, AA, GP, and PL	Spanish DCH (10 m)	[120]
II, IL, IV, LI, and LL	Spanish DCH (9 m)	[40,88,136]
GA, GP, and PG	Spanish DCH (9 m) Spanish DCH (6, 12, 18, and 24 m) In silico	[88,100,140]
VD, VDY, WK, VV, IE, and SI	Iberian DCH (24 m)	[122]
VF	Spanish DCH (18 and 24 m)	[87]
AS, QN, and YA	Laowo DCH (12, 24, and 36 m)	[137,141]
AD, EA, PE, PP, and VE	Iberian DCH (24 m)	[122]
STY	Spanish DCH (9 m)	[88,120]
***Immunomodulatory activity***		
LL, RL, and EKL	Spanish DCH (9 m)	[133,142,143]
AL and VH	Spanish DCH (9 m) Spanish DCH (12 m)	[3,69,87,120]
QPL, EK, YP, DLE, LGD, DSN, EAD, AAP, LGT, TGL, GQP, LV, PE, MV, LAP, LM, IGA, LTN, MSL, and ENP	Panxian DCH (36 m)	[144]
AQ	Spanish DCH (2 m)	[22,89,145]
γ-EF, γ-EI, γ-EL and γ-EY	Parma DCH (18 and 24 m)	[81,92]
γ-EF, γ-EW, and γ-EY	Prosciutto DCH (14, 21, and 34 m)	[93]
***Lipid metabolism-modulating activity***		
KA and VK	Spanish DCH (2 and 9 m)	[86,91,133,134]
DA, DD, EE, ES, and LL	Spanish DCH (6, 12, 18, and 24 m)	[87,146]
***Brain health promoting and neuronal-related activities***		
γ-EW	Parma DCH (18, and 24 m)	[81,92]
	Prosciutto DCH (14, 21, and 34 m)	[93]
HK, HP, LR, and VY	Spanish DCH (12 m) Jinhua DCH (8 m)	[3,22,83,133]

^a^ Peptides are given in one-letter code. DCH indicated dry-cured ham. ^b^ Activities given in italics.

### 8.1. Antihypertensive Activity

Cardiovascular disorders have become the main causes of world-wide death promoted by human bad habits and related pathologies [147]. The renin–angiotensin–aldosterone system is a set of routes to control the homeostasis of body fluids, mainly blood pressure and fluid homeostasis. The conversion of inactive angiotensin-I to angiotensin-II is catalyzed by ACE-I, and its activity constitutes a determinant role in cardiovascular health, since angiotensin-II is a potent vasoconstrictor and a risk factor of higher blood pressure [148]. ACE-I is an exopeptidase that cleaves dipeptides from the C-terminus. It is a chloride-activated zinc metallopeptidase, and it is assumed that the function of the anion activation in ACE-I provides high in vitro substrate specificity [128].

ACE-I-inhibitory peptides are mostly constituted by hydrophobic amino acids, with a low isoelectric point (3 to 6) and with a higher frequency of aromatic or alkaline amino acids in N-terminal position [116]. The enzyme has a strong substrate specificity and is influenced by the C-terminal tripeptide sequence of the substrate. A hydrophobic nature of the three C-terminal positions, such P, K, or R residues, improve the possibilities of interaction [149]. ACE-I competitive inhibition studies using dipeptides revealed residues V, I, A, R, Y, and F, and residues W, Y, F, P, I, A, L, and M are preferred in penultimate and ultimate positions, respectively, demonstrating a specificity by the last two localizations. Quantitative structure–activity relationship (QSAR) modelling of food-derived ACE-I inhibitory dipeptides showed that amino acid residues with bulky side chains as well as hydrophobic side chains such as F, W, and Y are preferred. For tripeptides, the most favorable residues for the C-terminus are aromatic amino acids, while positively charged amino acids are preferred for the middle position, and hydrophobic amino acids, in the N-terminus. The amino acid W is of high importance since the residue is located within the hydrophobic core of the ACE-I active site. Docking studies have revealed that P and Y in the C-terminus dock into the hydrophobic core of ACE-I [148]. Other QSAR modelling studies have predicted ACE-I inhibitory di- and tripeptides probably derived from food-proteins. The majority of di- and tripeptides contained W in the last position, highlighting the relevance of this residue and by extension other aromatic residues in C-terminal position [150,151].

Water-soluble peptide extracts fractionated by size-exclusion chromatography from pork dry-fermented sausages have exhibited ACE-I-inhibitory activity. Dipeptides AG, EG, EK, and KF identified from such fractioned extracts are potentially antihypertensive according to the BIOPEP database [13]. Actually, there is an important influence of the starter culture and protease addition on the bioactive capacity of dry-fermented sausages [152].

Different approaches have demonstrated dry-cured ham derived peptides have anti-hypertensive potential [76,107,124,153]. RPR, a tripeptide generated from nebulin in simulated gut conditions of pork meat, showed significant antihypertensive activity after oral administration to spontaneously hypertensive rats [91,124]. Dipeptides, such as AA, GP, KA, RP, and VY, and several tripeptides generated in dry-cured ham could exert ACE-I inhibition [88]. AA and KA were catalogued to be probable to be generated in dry-fermented sausages [7]. Actually, the dipeptide AA, quantitated along the processing of Spanish dry-cured ham and in low-salted samples, has recently been determined to act as a potential hypotensive, by in vitro inhibition of ACE-I and by oral administration to SHRs [90]. In addition to VY being found as bioactive in BIOPEP, the same peptide found in sardine muscle hydrolysates was tested in mild hypertensive subjects, appearing to have a significant antihypertensive effect via ACE-I inhibition, as well as in spontaneous hypertensive rats, with a prolonged reduction of systolic blood pressure, but no adverse effects could be detected at all. This dipeptide was found to reduce tissue ACE-I activity and angiotensin II levels in the abdominal aorta as well as in the kidney [154]. The activity of some dipeptides, AA, GP, KA, and RP, could be corroborated through a previous in vitro study of ACE-I inhibitory dipeptides generated by pork-isolated DPPs. Other peptides were assayed, and while RR exhibited a weak inhibition, GR and AR presented a moderate inhibition. AA, GP, and KA showed a higher inhibition, and RP was the strongest inhibitor. Interestingly, the dipeptides with higher inhibition could be generated through the action of DPP-II and DPP-IV [155]. The fact that DPP-IV remains active during the dry-curing process is a promising prospect regarding the generation of more dipeptides showing ACE-I inhibition in dry-cured ham. Tripeptides such as AKK, PAP, SGP, and TNP, highly probable to be generated from muscle proteins, also could exert antihypertensive potential according to the BIOPEP database [11]. Furthermore, a strong inhibitory tripeptide, LGL, and a less inhibitory sequence, ALM, were identified in Parma dry-cured ham 137]. In addition, some dipeptides derived from an in silico digestion of longer peptides identified in Iberian dry-cured ham have been reported to be potential ACE-I inhibitors. Some examples are EW, IF, GA, PL, and VF [122]. In fact, GA and VF have been detected in low-salted and traditionally elaborated Spanish dry-cured hams [87,100]. Additionally, the umami peptides EL, EV, RL, EEL, and ESV, identified in Jinhua dry-cured ham, have been reported with moderate in vitro ACE-I inhibitory activity [15].

There is more than one mechanism by which antihypertensive peptides can exert their effect. Renin is an enzyme that catalyzes the first step of the renin–angiotensin system, converting angiotensin I from angiotensinogen. Then, inactive angiotensin I is processed by ACE-I. QSAR, molecular dynamics simulation, and binding free energy analysis have revealed that renin inhibitory peptides establish hydrophobic forces and van der Waals contacts at the N-terminus of the peptide. Therefore, hydrophobic and/or bulky amino acids such as L, A, P, V, and W are preferred [156]. In agreement with those findings, partial least squares regression methodologies suggested that low molecular size amino acids with hydrophobic side chains are preferred at the N-terminus of inhibitory dipeptides while amino acids with bulky side chains are preferred at the C-terminus for potency. Finally, four W-containing antihypertensive dipeptides (IW, LW, VW, and AW) were predicted as the most potent renin inhibitors [157].

Peptide LR could present hypotensive activity in view of ACE-I and renin inhibition, whereas NR and EF would only act as renin inhibitors [91]. Another ACE-I and renin inhibitor is the dipeptide YA, found in Laowo dry-cured ham [137,157,158].

Renin inhibitors represent an alternative to ACE-I inhibitors; however, since renin and ACE-I form rate-limiting steps in this cascade and angiotensinogen is their only known substrate, simultaneous inhibition of these two steps would be a promising antihypertensive strategy [156].

**Figure 2:**
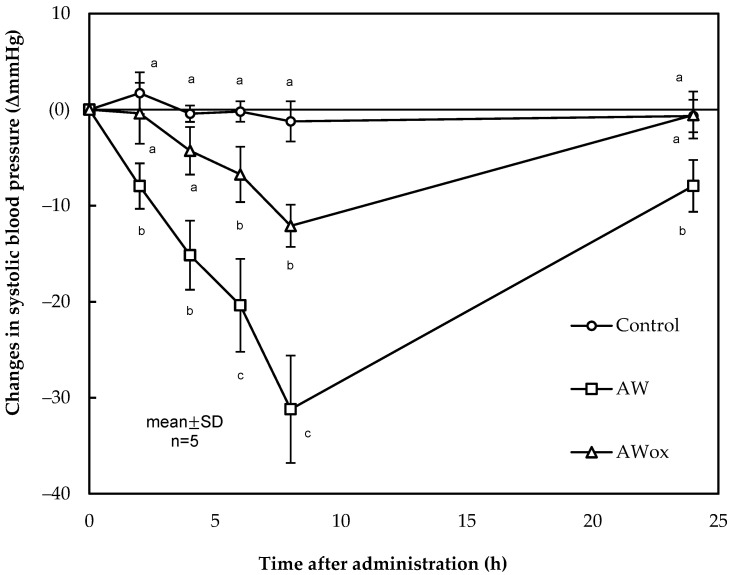
Systolic blood pressure drop with the treatment of a single oral administration of the dipeptide AW and AWox. Each point indicates the mean of systolic blood pressure of *n* = 5 SHR. Treatments were control (distilled water) and dipeptides AW and AWox. Different letters indicate significant difference from control at each time (*p* < 0.05). Reproduced from [85] with permission.

On the other hand, it is important to consider that during the processing time several post-translational modifications can occur, such as oxidation. Little investigation has been carried out until now on oxidized peptides as a consequence of the processing of dry-cured ham [13,159], but much less is known about the post-translational modifications of di- and tripeptides during the elaboration. However, these physicochemical reactions can alter the structure of the peptides, constituting potential modulators of their bioactivity and sensory properties. In this sense, the dipeptide AW has been found to accumulate to 5.12 μg/g dry-cured ham, and its oxidized form to 6.80 μg/g dry-cured ham in 12-months low-salted Spanish dry-cured hams. Both peptide forms exerted excellent in vitro ACE-I inhibition and hypotensive effects when orally administered on SHRs. However, the oxidized form exhibited less antihypertensive activity [85]. Figure 2 makes visible this data.

Various studies combine anti-hypertensive assays with antioxidant assays. The link between antioxidative and antihypertensive activities could be explained by the involvement of reactive oxygen species in renal injuries that can affect both renin–angiotensin and kallikrein–kinin systems, thus leading to hypertension [160].

### 8.2. Antioxidant Activity

Currently, a large part of the human population lives in environments affected by pollution and radiation, which together with bad habits and intrinsic conditions, such as aging, promote the exposition to reactive free radicals. Pro-oxidative stimulus causes cell damage and inflammation, leading to a high risk of developing chronic diseases including cardiovascular diseases, type II diabetes, hypertension, and obesity. In other hand, in terms of food technology and meat quality, bioactive peptides could suppress the oxidative deterioration of meat and products during refrigeration storage [161,162].

The well-accepted in vitro methodology to detect antioxidant compounds focuses on the hydrogen atom transferring, single-electron transferring, or metal ion chelating ability of the target substrate [163,164]. Hydrogen atom transferring-based assays include the inhibition of low-density lipoprotein autoxidation, oxygen radical absorbance capacity (ORAC), total radical trapping antioxidant parameter (TRAP), and crocin bleaching assays. In contrast, single-electron transfer reaction assays involve total phenols assay by Folin–Ciocalteu reagent, Trolox equivalence antioxidant capacity (TEAC), ferric ion reducing antioxidant power (FRAP), total antioxidant potential assay using a Cu(II) complex as oxidant, and DPPH radical scavenging assay [163].

Peptides can have distinct activities in different assay systems, but several combinations of amino acids can have synergistic effect with some antioxidant assays. Antioxidant peptides are generally characterized by a low isoelectric point. The sequence is known to play an important role in free radical scavenging effect. Compared with large molecular peptides, the small peptides under 5 kDa have better scavenging effect on 2,2-diphenyl-1-picrylhydrazyl (DPPH). The scavenging rate becomes lower as the chain of peptides increases its length due to the hydrophobic effects of amino acids [165].

The frequency of amino acids such as Y, E, and D is correlated with the antioxidant activity. Concretely, having an L residue in the N-terminal location could enhance the interaction with lipid free radicals [116], since hydrophobic amino acids such as A, I, L, P, F, Y, and W have been described to be able to increase the presence of peptides at the water–lipid interface and then access to scavenge free radicals from the lipid phase. Aromatic residues such as Y, W, and F can donate protons to an electron-deficient radical, contributing to the radical-scavenging properties, while the imidazole group of the H residue has good lipid peroxyl radical trapping capacity. In this line, antioxidant in vitro studies have revealed that the amino acids Y, W, C, or M contained in antioxidant dipeptides increase the radical scavenging activity, whereas W, Y, or C amino acids at the N-terminal position in a tripeptide are favorable residues for antioxidant capability [148,163]. For example, WV and VW have demonstrated a radical scavenging activity, and CW has been qualified as a potent antioxidant peptide [166]. Particularly, the content of H and its presence in the N-terminal position correlates with the antioxidant activity. This may be explained by the imidazole ring, with hydrogen donating ability, lipid peroxyl radical trapping, and metal ion-chelating ability [164]. In addition, acidic amino acids utilize carbonyl and amino groups from the side chain functioning as chelators of metal ions [128].

Porcine myofibrillar proteins are a good source of potential antioxidant peptides, as hydrolysates obtained by protease treatment possessed high antioxidant activity in a linolenic acid peroxidation system induced by Fe^2+^, DPPH radical scavenging activity, and chelating activity toward metal ions. Likewise, the acidic fraction obtained by ion exchange chromatography exhibited higher activity than the neutral or basic fractions. The major constituent amino acids in porcine myofibrillar proteins are E, D, and K (15, 9.5, and 8%, respectively). These amino acids may interact with metal ions through their charged residues and inactivate prooxidant activity of metal ions [162]. In this sense, water-soluble peptide extracts fractionated by size-exclusion chromatography from Spanish, Italian, and Belgian porcine dry-fermented sausages have exhibited antioxidant activity. It is known that despite the fact that peptides are generally more effective as antioxidants than free amino acids, they can also contribute to the bioactivity profile. In fact, hydrophobic amino acids such as A, F, V, P, G, L, and I were present in significant amounts in the assayed dry-fermented sausages and might be also contributing to the antioxidant activity. A dipeptide was identified from the fractions, IY, which has antioxidant activity according with BIOPEP database [167]. Time-dependent proteolysis observed in fermented pork meat sauce during the fermentation period increases DPPH radical scavenging activity, which remains higher than the activities of sauces from other animal sources. What is more, high antioxidant tripeptide QYP has been detected in fermented pork meat sauce, and it has been suggested that it comes from troponin, connectin, myoplasm protein, or muscle stroma protein [168]. As in the generation of potential antihypertensive peptides, of particular interest is that the starter culture and protease addition to dry-fermented sausages has been demonstrated to have a dominant role on the generation of antioxidant compounds [152]. On the other hand, porcine blood is an important by-product in the meat industry and considered to be a potential source of nutritional and functional protein source. For instance, porcine plasma protein hydrolysate with alcalase can play an antioxidant role in the radical-mediated oxidation system [169]. Hydrolysates from porcine bone extracts also seem to be a good source of antioxidant short peptides [105].

Dry-cured ham-derived peptides have exerted alleviative effects on the generation of reactive free radicals [76,107,123,170,171,172]. Small peptides AY, EL, KP, VY, and EAK are probably generated in dry-cured ham and according to BIOPEP database, they could exert antioxidant activity [91]. Particularly, the AW dipeptide, quantitated in dry-cured ham, has demonstrated high in vitro antioxidant bioactivity, while its oxidation seemed to benefit the ET-based methods DPPH free radical scavenging assay and Ferric reducing power [85].

In addition, the peptides SY, PN, GS, and KP were proposed to be generated in antioxidant hydrolysates (<1 kDa) from bone peptide extracts, while the peptides QPL, EK, YP, DLE, LGD, DSN, EAD, AAP, LGT, TGL, GQP, LV, PE, MV, LAP, LM, IGA, LTN, MSL, and ENP, were identified in the most antioxidant fraction (below <3 kDa) from Panxian peptide extracts [144].

Histidine-containing dipeptides carnosine and anserine are widely distributed in skeletal muscle of pork among other species and constitute important antioxidants acting as pleiotropic chemicals [173]. They have protecting effects, blocking reactive species generated during oxidative metabolism, buffering the excess of hydrogen cations accumulated during non-oxidative processes, and are implicated in the regulation of calcium required for muscle contraction. H-containing dipeptides’ imidazole ring makes them biochemically competent to act as buffers in skeletal muscle metabolism and specifically in anaerobic routes, by which a higher buffering capacity is required. For instance, carnosine content in type II muscle fibers is significantly higher than their type I counterparts. In fact, β-A supplementation promotes human endogenous synthesis of carnosine, which has an ergogenic effect in high-intensity exercise tests. Other purported roles include metal chelation, anti-glycation, or anti-carbonyl activities [174].

Oxidative cellular damage comprises a wide variety of mechanisms in which reactive carbonyl species such as advanced glycation-end products and advanced lipid oxidation-end products are involved [175]. These compounds have been accepted as biomarkers for oxidative-based diseases and are generated by sugar derivatives and through the oxidation of lipids, respectively. For this reason, carbonyl scavengers able to trap reactive carbonyl species represent an interesting area to transform them into non-toxic and excretable derivatives and inhibit in vivo protein carbonylation [176]. Studies have suggested that supplementation with H amino acid or H-rich peptides reduces the production of these abducts [175]. This is the case of the dipeptide carnosine, which can reach 300 mg/100 g of *M. semimembranosus* in dry-cured ham and, although it cooperates with the bioactive peptide derivative anserine in reducing rancid taste and improving color stability [177], it has been identified as a quencher of these reactive species by in vitro and in vivo approaches. However, carnosine is a target of serum and tissue carnosinases and its bioactivity is limited [178]. Thus, dipeptide analogues with similar properties have been studied, finding that aromatic and positively charged residues and ester derivatives benefit quenching phenomena. Meanwhile, shortening of the C-terminus and a greater rigidity result in a detrimental effect on quenching. Dipeptides such as KH-X, (X = OMe or NH2), CH-X, YH-OMe, and W-containing dipeptides have remarkable quenching bioactivity and a higher stability than carnosine [176].

Based on these findings, proteolysis in porcine dry-cured meat could generate dipeptides with detoxifying effects.

### 8.3. Antidiabetic Activity

Currently, diabetes is one of the main chronic diseases with the greatest impact on mortality, along with cardiovascular diseases, cancer, and chronic respiratory diseases [147]. Glycemic control in diabetes mellitus is essential for monitoring and therapeutic regimen.

In response to nutrient ingestion, incretin hormones glucagon-like peptide (GLP-I) and glucose-dependent insulinotropic peptide (GIP) are respectively released mainly by L-cells located in the distal ileum and by enteroendocrine K-cells in the proximal gut. Incretins promote the release of insulin by the pancreas, the suppression of glucagon secretion, and lessen the glucose blood levels. However, this signalization can be interrupted by the enzyme DPP-IV, a serine protease expressed ubiquitously. It cleaves preferentially XP or XA dipeptides from the N-terminus of multiple substrates, including incretin hormones before leaving the gastrointestinal tract. For this phenomenon, there are therapies based on the usage of agents that inhibit DPP-IV, which promote the increase of active levels of these hormones and improve islet function and glycemic control in diabetes. Another therapy consists of injection of GLP-I agonists, but it is still limited by its short half-life because of its rapid breakdown by DPP-IV [179,180].

DPP-IV has been described to possess various inhibitor binding sites, one at the active site and another one near this last. Most of the previously reported DPP-IV inhibitory dipeptides have been analyzed using porcine DPP-IV, but the identity of the primary structures between human and porcine enzymes is very high (88%). Therefore, the inhibitory effect of dipeptides for porcine DPP-IV should be similar to that for human DPP-IV [181]. W-containing dipeptides play a role as DPP-IV inhibitors and modulators of oxidative stress. Except for WD, dipeptides with a W residue at the N-terminus have been probed to act as in vitro DPP-IV inhibitors. Particularly, dipeptides WR, WK, WL, and WP exert a strong DPP-IV inhibition. Fewer dipeptides containing W at C-terminus LW, MW, and AW were found to be DPP-IV inhibitors. WL and LW behave as competitive inhibitors, while the rest, WR, WK, WP, WA, WQ, WI, WN, WM, WK, WC, WT, WW, and WS, exert a non-competitive inhibition [166]. In agreement with these findings, a systematic analysis based on DPP-IV specificity determined that dipeptides WP and WA, and suddenly, WP and WR, exhibited the highest competitive-type inhibitory effect. Conversely, tripeptides starting by WR presented less inhibition, but all of them showed unique uncompetitive-type inhibition, suggesting these tripeptides as lead sequences for DPP-IV inhibitors with a mechanism of action different to that of dipeptides [141]. These findings suggested that residues at the N-terminus determine the inhibitory potency and that the inhibitory effect of dipeptides is higher than that of tripeptides. Additionally, dipeptides composed by aromatic amino acids with polar sidechain and P at the N-terminus, derived from dietary proteins, were deduced from sequence alignment of different DPP-IV inhibitory peptides. Finally, a hydrophobic N-terminal amino acid such as W, I, F, and L, in a dipeptide was predicted to exert inhibitory bioactivity [148]. On the other hand, an in vitro study of the inhibitory activity of a set of commercially available dipeptides on human DPP-IV demonstrated that TH, NH, VL, ML, and MM were strong inhibitors [181].

IPI (diprotin A) is a well-known tripeptide that acts as a potent inhibitor of DPP-IV. Molecular docking analyses have revealed sequences such as APA, APF, APR, IPA, KPA, FPF, FPI, FPW, IPF, IPW, WPF, WPT, and WPW, which are described to possibly act as IPI analogues, can bind to different potential DPP-IV binding sites [182].

DPP-IV inhibitory short peptides have been identified from animal food protein hydrolysates, specially from milk proteins [181,182]. In contrast, less is known about DPP-IV inhibitory peptides present in dry-cured ham [120].

Collagen hydrolysates constitute potential sources of bioactive peptides. Specially, collagenase-treated pigskin collagen hydrolysate has been probed to exert in vitro DPP-IV inhibitory activity. The polypeptide chain of this protein is composed by GP-Hyp (Hyp = hydroxyproline) tripeptide units but also of GPA, GA-Hyp, and GL-Hyp, and collagenase treatment releases these tripeptides, which exert DPP-IV inhibitory potential. Of the three peptides, GP-Hyp constitutes a true peptide inhibitor of the enzyme, because DPP-IV cannot hydrolyze the bond between P-Hyp and this tripeptide was found to be a moderately competitive inhibitor [183].

An in silico approach was carried out to analyze the potential of selected pork muscle proteins to generate bioactive peptides with anti-diabetic properties, basing on the frequency of bioactive fragments of the tested activity in the protein chain and peptide affinity for the specific receptor characterizing the potential activity. All selected proteins were potential sources of DPP-IV inhibitors and of glucose uptake stimulating peptides as a result of in silico digestion by pepsin, trypsin, and chymotrypsin. Many dipeptides such as AL, AY, DR, EK, PK, PL, QL, SK, TL, VF, VK, and VY were found as potential DPP-IV inhibitors and IL also as glucose uptake stimulators [184].

The most frequent bioactivity of probable small peptides generated in dry-cured ham was DPP-IV inhibition [88]. Dipeptide KA was previously assayed with a remarkable DPP-IV inhibitory activity (IC_50_ value of 6.27 mM), and weaker IC_50_ values were reported for AA, GP, and PL [120]. In addition, elucidated dipeptides, II, IL, and LL from dry-cured ham proteins could exert positive effects on glucose regulation by both DPP-IV inhibition and stimulating glucose uptake activity. Dipeptides containing branched-chain amino acids such as II, IL, IV, LI, and LL have been reported to present this activity possibly via kinase signaling pathways, which are different from the mechanism of the insulin-stimulated glucose transporters [88]. An in silico approach determined that GA, GP, and PG were the most frequently occurring sequences within dietary proteins, with previously described DPP-IV inhibitory activity [140]. Interestingly, among them, GA was reported to probably be released during proteolysis in dry-cured ham [91], which was then quantitated in Spanish low-salted dry-cured hams [100]. Other potential DPP-IV inhibitors described to be generated during Iberian dry-cured ham processing are VD, VDY, WK, VV, IE, and SI [122]. Another example is VF, which was also identified in dry-cured ham, as pointed out above [87]. On the other hand, AS, QN, and YA were found in Laowo dry-cured ham [137], with previously demonstrated DPP-IV inhibitory properties [181].

Located in the brush border membrane of the small intestine, α-glucosidase enzyme complexes hydrolyze oligosaccharides into monosaccharides. Thus, inhibition of these complexes means delayed carbohydrate absorption and digestion, which results in a reduction in postprandial hyperglycemia. There are three currently available α-glucosidase inhibitors: acarbose, miglitol, and voglibose, and they act as structural analogues of natural oligosaccharides with higher affinity for α-glucosidases, but unlikely, they exert numerous side effects as gastrointestinal discomforts due to the fermentation of undigested carbohydrates by the microbiota [180]. For this reason, naturally occurring bioactive peptides are of interest as an alternative to prevent or palliate drug side effects.

Less is known about the structural requirements for α-glucosidase inhibition; however, several hypotheses have been reported basing on already described food-derived α-glucosidase inhibitory peptides. A sequence from three to six residues with either S, T, Y, K, or R at the N-terminus and a P residue closer to the C-terminal with residues M or A occupying the ultimate C-terminal position, could attribute inhibitory potential [148].

A clinical study has reported that daily consumption of 80 g of Spanish dry-cured ham had a hypoglycemic effect, dropping fasting blood glucose levels [185]. In agreement with these results, various potential α-glucosidase inhibitory peptides from Spanish dry-cured ham, for instance AD, EA, PE, PP, and VE, have been evaluated. Interestingly, dipeptides EA, PP, and VE were previously described as ACE-I inhibitory peptides, whereas dipeptides PP, VE, PE, and AD were described as DPP-IV inhibitory peptides, which demonstrates their multifunctional potential [186]. Chromatographic approaches have evidenced that most α-glucosidase inhibitory peptides generated in dry-cured ham are polar, and in silico digestion of the identified peptides with a Peptide Ranker value higher than 0.5 has allowed to detect previously described ACE-I, DPP-IV, and DPP-III inhibitory dipeptides [122].

Molecular docking studies suggested that STY, a tripeptide resulting from an in silico simulated gastrointestinal digestion of a potent α-glucosidase inhibitor STYV, presented hydrogen bonding interactions and binding energies comparable with acarbose [184]. These findings allow to predict that STY will have high α-glucosidase inhibitory activity, and it has been described to probably be present in dry-cured ham [88].

### 8.4. Immunomodulatory Activity

Obesity, type-2 diabetes, and cardiovascular pathologies, which are leading causes of mortality worldwide, are highly correlated with chronic inflammatory conditions. Unbalanced pro-inflammatory signalizations can cause tissue damage and a deficiency on the immune function [131].

The study of immunomodulatory peptides is highly complex because the target is relatively nonspecific and the exact mechanism of their actions as well as their in vivo metabolism is largely unknown, as well as their structure characteristics. Most of the studies have been conducted using cells of the specific and unspecific immune system. Many food-derived peptides exhibit their anti-inflammatory activities primarily by inhibiting signaling components of either NF-κB or MAPK pathway, which are the two major pathways involved in chronic inflammation following uncontrolled signal activation. Peptides with immune functions are usually short, hydrophobic, and cationic, especially in the N- and C- terminal [130,131]. However, inconsistent findings have also been reported. The global positive charge may act as a chemokine, while the presence of R in the sequence and in the N- and C-terminals benefit the anti-inflammatory activity. Additionally, a basic N-terminal has been reported to be able to chelate lipopolysaccharide (LPS) and block the LPS-induced inflammation response. In addition, the frequency of Q residues in the sequence could reduce LPS-induced prostaglandin D2 and nitric oxide production in RAW 264.7 macrophage cells. The presence of P confers resistance to intestinal digestion, which with a hydrophobic nature of the residues could improve the ACE-I inhibitory and anti-inflammatory activities. Specially, it has been reported that highly hydrophobic amino acids situated in the N-terminal, while polar groups are located at the C-terminal, could exert anti-inflammatory activities [131].

Regarding meat-derived bioactive peptides, few studies have ever focused on the interrelation between ACE-I inhibitory activity and anti-inflammation response as well as the involvement of cell signaling pathway. ACE products angiotensin II and angiotensin III are involved in chemotaxis and adhesion of monocytes and macrophages and in the activation of transcription factors NF-kB and AP-1. Thus, antihypertensive peptides could have an immunomodulatory effect [160]. Recently, it has been demonstrated that peptides generated in Spanish dry-cured ham can exert both potential ACE-I inhibitory and anti-inflammatory in vitro bioactivities, but such promising results need to be supported with further approaches as well as its mechanism studied for a better understanding of efficacy and bioavailability [76].

Food-derived bioactive peptides have been mostly reported to modulate ex vivo the production of proinflammatory mediators in macrophages by decreasing the production of free radicals, proinflammatory interleukins, and prostaglandins [130]. Although there is little evidence about immunomodulatory peptides generated in pork meats, less is known about naturally generated di- and tripeptides with a potential role on immunity.

Interestingly, mammalian members of the proton-coupled oligopeptide transporter family, such as PepT1 and PepT2, are integral membrane proteins that mediate the cellular uptake of dipeptides, tripeptides, and peptide-like drugs [187]. PepT1 is expressed in immune cells, including macrophages, and regulates the secretion of proinflammatory cytokines triggered by bacteria and/or bacterial products, with a key role in intestinal inflammation [188]. In this sense, anserine, a bioactive peptide from skeletal muscle, has been suggested to be probably transported by Pept1 [178]. This means peptides could target immune cells via their transport.

**Figure 3:**
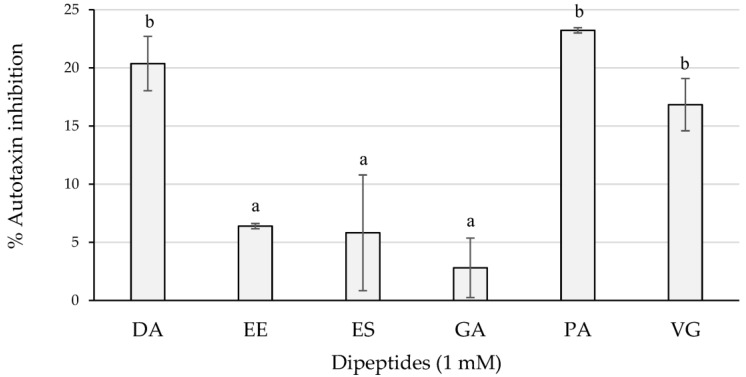
Autotaxin inhibition percentages of the dipeptides DA, EE, ES, GA, PA, and VG (DG was null), identified in 12 months dry-cured ham extracts with low-salt content, at 1 mM (*n* > 3). Different letters indicate statistically significant differences (*p* < 0.05) between inhibitory activities. Reproduced from [100] with permission.

Boiled pork meat and hot water extracts of pork meat have been found to act as antioxidant agents and inhibit secretions of inflammatory cytokines by decreasing TNF-α and IL-6 in RAW 264.7 cells, while water extracts of pork loin and ham improved the viability of LPS-induced RAW 264.7 cells [173]. Conversely, another study suggested that both types of extracts may stimulate the production of several cytokines secreted by splenocytes from BALB/c mice, manifesting pork meat could be regarded as an immunostimulatory agent. However, mice fed with high concentration of hot water extracts of pork meat experienced a decrease in IL-2/IL-4 secretions when stimulated with concavalin A and a reduction in TNF-α/IL-10 secretions when treated with lipopolysaccharide. Thus, high concentration of hot water extracts of pork meat would have anti-inflammatory effects on the primary splenocyte. This variety of results can suggest the wide range of action of derived-protein compounds as immunomodulators. Some in silico approaches, based on the amino acid sequences of empirically validated anti-inflammatory epitopes and non-anti-inflammatory epitopes and linear peptides, could be used to detect potential anti-inflammatory peptide compounds [142,143]. Among them, potential anti-inflammatory peptides LL, RL, and EKL are probably generated in dry-cured ham [22]. Dipeptides AL and VH, identified in pepsin–pancreatin hydrolysate of velvet antler protein, and also cited as potential regulators of glycaemia [91], exhibited anti-inflammatory activities by inhibition of NO production in LPS-induced RAW 264.7 macrophages [69]. Actually, AL has been identified in Spanish dry-cured ham samples [87]. In this line, several other dipeptides, quantitated in Spanish 12 months-aged dry-cured hams elaborated with less salt, namely DA, EE, ES, GA, PA, and VG, have been reported to exert in vitro inhibitory activity on various pro-inflammatory enzymes. Figure 3 replicates the in vitro inhibition activities at 1 mM for autotaxin.

On the other hand, QPL, EK, YP, DLE, LGD, DSN, EAD, AAP, LGT, TGL, GQP, LV, PE, MV, LAP, LM, IGA, LTN, MSL, and ENP, were found in the most PAF-AH inhibitory fraction (below <3 kDa) from Panxian peptide extracts [144].

Intriguing findings have been obtained about the Q amino acid. It is a recognized precursor of protein synthesis, functions as intermediate in various pathways, and serves as nitrogen transporter and as regulator of amino acid homeostasis [189]. It serves as an energy source for dividing immune cells and it is beneficial for clinical outcomes [190], but at the same time, it presents low solubility, which constitutes an impediment when designing supplements or administering it by parenteral nutrition solutions and preparation procedures become more complex. However, dipeptides with Q residue in C-terminal position, such as GQ and AQ, present better physicochemical properties and can be used as carriers of this amino acid because they are rapidly hydrolyzed [145,189]. Numerous studies corroborate the application of dipeptides as carriers of this amino acid.

The dipeptide GQ has been reported to improve the status of the gastrointestinal tract by increasing Q amino acid consumption, which boosts cell proliferation and inhibits cell apoptosis. Supporting results were obtained in an in vitro study in jejunal tissues from weaned piglets, noticing that GQ dipeptide promotes Q-related enzymatic synthesis, jejunal cell proliferation, but L-lactate dehydrogenase, a marker of cell death, and caspase III generation are reduced [190].

On the other hand, severe burn injury and subsequent major eschar excision can lead to increased gut permeability and enhanced plasma endotoxin levels, with a deficiency in Q serum concentrations, which is known to be essential in immunity and gut integrity. It has been demonstrated that AQ dipeptide supplementation can reduce the infection rate, wound healing time, intestinal permeability, and serum endotoxin concentration at the same time that plasma Q levels are increased, and the status of the patients is improved [191].

Consistent with these results, AQ supplementation in rats attenuate inflammatory effects of resistance exercise by decreasing the cytokine levels and reduction of DNA-binding activity of NF-kB in extensor digitorum longus muscle. HSP70 was seen to be increased in both peripheral blood mononuclear cells and muscle and a decline in TNF-α, IL1-β, creatine kinase, and lactate dehydrogenase was perceived in plasma, as well as an increase in IL-6, IL-10, and MCP-1. Finally, a restoration of Q levels in plasma and muscle was reported [192]. In addition to this, it has been proved in piglets that AQ supplementation can upregulate protein synthetic routes in liver and skeletal muscle via activating the rapamycin signaling pathway, at the same time that there was a decrease in protein degradative signaling via downregulating ubiquitin–proteasome proteolysis pathways [193].

Furthermore, AQ, as well as GQ, decrease the release of pro-inflammatory cytokines by polymorphonuclear leukocytes, while expression of the anti-inflammatory IL-10 is enhanced [189].

Nowadays, Q-containing dipeptides are an integral part of the routine in clinical practice in order to re-establish intracellular muscle free glutamine pool, increase of protein synthesis, boost glutathione production and lymphocyte count, maintain gut and intestinal functions, minimize infectious complications and on balance, length of hospital stay and mortality [189]. The fermentative production is a way for obtaining the AQ dipeptide [145], but it has been estimated to be present in dry-cured ham [22]. Hence, this product might be a good source for natural supplementation.

Such results evince that di- and tripeptides can serve as delivering vehicles of functional amino acids, improving the pharmacokinetic properties to facilitate the bioavailability. Once ingested, amino acids would be released by digestive, intestinal, and/or serum peptidases and absorbed. For this reason, hydrolysis of small peptides can be taken in advantage to benefit, but it is normally a phenomenon that limits the effects of bioactive peptides. In this sense, γ-glutamylation of peptides may be a protecting reaction from further proteolysis, as they can only be hydrolysed by γ-glutamyl cyclotransferase [111].

The CaSR Is known to be distributed throughout the gastrointestinal tract and plays different roles via interaction with analogues. Glutamyl peptides have been reported to stimulate the secretion of parathyroid hormone from normal human parathyroid cells by modulating this receptor [111] but also, allosteric ligand activation of the CaSR by γ-EC and γ-EV can reduce inflammation in chronic inflammatory conditions like inflammatory bowel disease and intestinal inflammation. The in vitro treatment with these two peptides reduces pro-inflammatory cytokine production in Caco-2 cells. Simultaneously, these peptides ameliorate clinical signs and decrease the generation of cytokines in a mouse model of dextran sodium sulphate-induced colitis, apparently, by blocking activation of the TNF-α-dependent pro-inflammatory signaling cascade through TNF-α receptor. Actually, γ-EV supplementation has been shown to exert similar effects in dextran sodium sulphate-induced colitis porcine model [194,195]

As it has been signaled in the Kokumi section, γ-glutamyl peptides γ-EF, γ-EI, γ-EL, and γ-EY have been quantified in Parma dry-cured ham [81,92], and γ-EF, γ-EW, and γ-EY were detected in Prosciutto dry-cured hams [93]. Actually, a greater amount of these peptides has been found in digested samples of dry-cured ham compared with non-digested samples, so it can be hypothesized that γ-glutamyl residues are bound to N-terminal amino acids in peptide chains and then released from them by enzymatic digestion [92].

These findings constitute promising prospects in the study of pork-derived short peptides as possible immunomodulators, meaning that porcine products can lead to a recovery from the pathological status.

### 8.5. Antimicrobial Activity

Naturally occurring antimicrobial peptides are molecules that are easier to be produced and can be more potent. These compounds are characterized by a high broad-spectrum activity, and the risk to develop resistance does not occur in the short term as it is supported by their persistence in nature over millions of years [119]. In addition, peptides with antimicrobial attributes could be added to food or a food matrix in order to assure food safety [160].

Cationic charged as well as bulky and lipophilic residues are necessary in an amphipathic structure of these compounds [196]. A high hydrophobicity allows a better interaction between the peptide and the cell membrane, which may contribute toward modulating the downstream signaling pathways and exhibiting the anti-inflammatory effect [131]. Their capacity to form channels or pores within the microbial membrane impairs the possibility for anabolic processes [195]. On the other hand, charged amino acids generally consist of a side chain of R or K. Cationic compounds are known to benefit the interaction with negatively charged membrane phospholipids [119]. Regarding bulk units, they are represented by an indol, phenol, or phenyl groups. Other studies suggest that the antimicrobial activity of peptides containing R is higher than those of peptides containing K, while peptides containing Y are more potent than those with either F or Y. P-rich peptides, and additionally, in W, R, and H, with a lack of secondary structure, have been correlated with a broad spectrum antimicrobial activity [196].

Porcine myofibrillar proteins are rich in short sequences which are ligands of bacterial permeases. Selective peptide-binding activity of oligopeptide permeases may affect cell envelope permeability restrictions and the nutritional strategy required for survival of bacteria in a specific environment [197]. Dry-cured ham is a ready-to-eat meat product, which can be sliced and packaged under vacuum as an extra protection barrier prior to distribution and commercialization. However, these post-processing actions could favor the contamination due to the development of pathogenic organisms such as *Listeria monocytogenes*. Antilisterial peptides of 5 to 18 residues, belonging to most active SEC-fractions from dry-cured ham, were reported. Six of them presented Y and two positively charged amino acids such as H or K in their sequences, and several of the identified peptides included the antimicrobial motifs KYR and RYH [119]. Long peptides can adopt an α-helical linear or circular structure organized in a β-sheet, which are essential considering the mechanism of action of active peptides against the microorganisms. The results derived from this study prove the presence of peptides naturally generated during the processing of dry-cured ham and their antilisterial potential as preservative during storage and distribution. No dry-cured ham-derived di- and tripeptides with antimicrobial properties have been found to date. Notwithstanding, the minimum antibacterial motif of cationic antibacterial peptides regarding charge, lipophilicity, and bulkiness is found to be surprisingly small [196]. Therefore, these findings open the opportunity for the identification of unknown natural short antibacterial peptides in dry-cured ham.

### 8.6. Other Activities

#### 8.6.1. Antithrombotic Activity

Diets which inhibit platelet activation and aggregation might reduce the risk of atherothrombosis. Many commercialized oral antithrombotic drugs rely on passive diffusion mechanisms to cross cell membranes and reach the bloodstream. This results in poor bioavailability and failure to reach their targets. Fortunately, taking advantage of the structural characteristics of the peptides, peptide analogues can be designed to be captured by the peptide transport system. This is the case of the antiplatelet aggregating and antithrombotic drug 3-(S)-1,2,3,4-tetrahydro-β-carboline-3-carboxylic acid, with poor bioavailability. Basing on the structural skeleton, W residue was used as surrogate (*W) to create dipeptide analogues by the introduction of residues to the 3-position of tetrahydro-β-carboline scaffold. The study reported an improvement of the absorptive transport of peptide analogues through Caco-2 cell monolayers, particularly promoted by polar charged residues better than bulky residues. Most in vitro bioactive dipeptides, K-*W, R-*W, suggested that basic positive charges benefit both antithrombotic bioactivities, as well as C-*W and M-*W, indicating sulfur-containing side chains also improve the antiaggregatory effect. However, rigid and bulky dipeptide analogues reduce the bioactivity [132].

Antithrombotic peptides can act as competitive inhibitors for fibrinogen, and thus, charged amino acids, particularly at the C-terminal, may influence their antithrombotic efficiency by different molecular mechanisms. Porcine myofibrillar proteins, especially titin, content antithrombotic sequences according to an in silico digestion with digestive enzymes [197]. Upon treatment of porcine *longissimus dorsi* muscle with papain, a bioactive peptide fraction with mean molecular weight of 2500 Da was identified, which demonstrated in vivo antithrombotic activity after oral administration to mice [198]. In this sense, a clinical study demonstrated that 4 weeks of regular dry-cured ham intake resulted in a significant decrease of P-selectin expression in platelets stimulated with adenosine diphosphate, opening up an interesting avenue for further research [125].

#### 8.6.2. Lipid Metabolism-Modulating Activity

Obesity and hyperlipidemia increase the risk of diabetes and cardiovascular diseases. Plasma cholesterol levels, especially low-density lipoprotein (LDL), are generally influenced by diet and cholesterol biosynthesis, uptake, and secretion. The mechanisms by which peptides can exert hypolipidemic activities might be explained, on one hand, by the presence of some potent hydrophobic sequences, which are known to bind bile acids mainly due to hydrophobic interaction with tetracyclic ring structures, contributing to increased fecal excretion of dietary cholesterol. On the other hand, hypolipidemic activities could be attributed to the inhibition of lipogenic enzymatic activities and modulation of the gene expression [138,160,199].

An interest in the cholesterol-lowering capacity of the peptides is taking relevance in research, although little information about the identification of short sequences is available to date. However, there is enough evidence about the potential beneficial effect of dry-cured ham’s peptide content on the cholesterol levels. Plasma cholesterol has been reported to decrease in rats fed with partially hydrolyzed pork meat, more than those fed with intact pork meat. Interestingly, the amount of protease used for the hydrolysates was inversely correlated with hypocholesterolemic effect, suggesting the involvement of bioactive peptides on regulation of cholesterol homeostasis [200]. In the same line, papain-hydrolyzed pork meat reduced plasma cholesterol, concretely the very low-density lipoprotein and the LDL, in rats treated with a cholesterol-enriched diet. On the contrary, higher cholesterol levels were found in rats fed with untreated pork meat or soybean protein and increased fecal excretion of neutral and acidic steroids [201]. Additionally, a low molecular weight fraction of papain-hydrolyzed pork meat exerted reduction of cholesterol levels and preventive effects of premature atherosclerosis in dietary-induced hypercholesterolemic rabbits [202]. More recently, the regular consumption of dry-cured ham was reported to drop total cholesterol and LDL [127]. Curiously, hydrophilic dipeptides KA and VK, probably generated in dry-cured ham [91], were identified in soy protein hydrolysate and reported to decrease triglyceride synthesis in cultured hepatocytes [86]. Moreover, dry-cured ham-derived dipeptides DA, DD, EE, ES, and LL have been characterized as potential hypocholesterolemic agents through in vitro and in silico HMG-CoAR inhibition [87,146].

Further to the LDL-lowering activity, evidence suggests that pork could provide short peptides with anorexigenic routes. Food intake regulation strongly relies on the gut–brain axis [203] and satiety hormone secretion is highly governed by food. During ingestion, peptide signals from the gut can change the attitude to food from the “hunger” state to the “satiation” state [179]. Proteins are recognized as the strongest inducers of satiety macronutrients, and high-protein diets have been correlated with reduction of total energy intake, body weight, and fat deposition. Organoleptic properties, influenced by food processing, contribute to the satiety induced by protein foods [103,104]. Appetite-suppressing bioactive compounds from foods offer an attractive alternative to pharmacological solutions to control appetite.

At first instance, amino acid composition of dietary proteins regulates appetite depending on threshold values. E amino acid could function as a marker of protein ingestion because it is the most abundant residue in almost all dietary proteins. L amino acid levels influence on energy sensors of the control of energy intake, at least in the arcuate nucleus of the hypothalamus. Finally, some amino acids such as Y and H could exert satiating effects through their role as precursors of neurotransmitters [104].

Dry-cured ham constitutes an excellent source of high-biological-value proteins because it contains essential amino acids in appropriate ratios and proteins are readily digestible. Protein content is about 30 g/100 g depending on the extent of drying and the fat content. Large amounts of free amino acids are generated in hams at levels of hundreds of milligrams per 100 g; for instance, K amino acid reaches near 700 mg/100 g [147].

Peptide transporters and receptors activate satiety hormones secretory mechanisms via the enteroendocrine cell membrane [179], concretely by GPCR C group 6 member A, the umami taste receptor T1R1/T1R3, the calcium-sensing receptor, the metabotropic glutamate receptors, and the peptide receptor GPR93 [204]. Taste receptors are distributed in the oral cavity, gastrointestinal tract, enteroendocrine cells, and β-cells in pancreas [28,30].

Ingestion of nutrients into the small intestine stimulates the anorexigenic peptide cholecystokinin (CCK) by enteroendocrine cells [205], and it is transmitted to the brain. Detection of amino acids and oligopeptides occurs in the upper intestine, within the lumen of the duodenum upon the release of this hormone [104]. Studies suggest that endogenous peptides, dietary proteins, and their digestion products bind to the rat small intestinal brush–border membrane to be detected by CCK-producing cells directly to stimulate CCK release [205]. In the lower intestine, another anorexigenic peptide, YPIKPEAPGEDASPEELNRYYASLRHYLNLVTRQRYX (PYY-1), is released with a subsequent inhibition of intake. In fact, PYY-1-knockout mice manifest obesity, which is reversed by exogenous PYY-1 administration [104]. Ghrelin is an orexigenic hormone known for its meal-initiating activity and whose levels respond in a compensatory manner to the energy deficit/excess. It is secreted from the fundus of the stomach. Other related gastrointestinal hormones are GLP-I, which via the central nervous system can suppress appetite, and GIP, both playing a role on the secretion of insulin [206].

Anorexigenic gut peptides and the resulting circulating amino acids transmit suppressing food intake signals directly or indirectly through hormonal mediators to the brain [104]. Pharmacological peptide solutions to appetite suppression include analogues of GLP-I and structural variants of CCK to confer enzymatic proteolytic resistance and are administered by injection rather than orally. However, the daily injection regime and common side effects of nausea, constipation, and diarrhea make them unattractive for long-term use [179]. For this reason, dietary proteins can be a source of encrypted bioactive peptides cleaved from by the action of digestive or brush border enzymes. Additionally, food-derived peptides generated by endogenous, microbial, and/or commercial enzymes can exert satiety bioactivity.

Interestingly, it has been reported that high-protein diets stimulate the release of PYY-1 in agreement with a pronounced satiety. High-protein meal ingestions also decrease the orexigenic peptide ghrelin and stimulate the anorexigenic inductors gastric inhibitory polypeptide and glucagon-like peptide I production [104].

The CaSR is one of the receptors involved in the release of GLP-I and it is preferentially activated by aromatic amino acids [203]. Peptone from an enzymatic meat hydrolysate and peptides GF, GL, and LGG have been demonstrated to stimulate GLP-I production in urine primary colonic cultures via PEPT I and CaSR [207]. Additionally, other transporters might be implicated [203]. Porcine skin gelatin hydrolysate has shown to inhibit plasma DPP IV activity and raised GLP-I and insulin levels in streptozotocin-induced diabetic rats [208]. K and N residues have been suggested to confer GLP-I releasing activity, and peptides triggering CCK release may be characterized by containing at least one aromatic amino acid residue in their sequences [203].

Reasonably, in cooperation with DPP-IV peptide inhibitors, a prolonged half-life of GLP-I could contribute to the release of insulin and reduction of appetite [203]. In the same line, high-protein diets have been reported to induce gluconeogenesis and prevent a decrease in glycaemia that could contribute to satiety [104], which also might be due to DPP-IV inhibition and consequent support of GLP-I and insulin signalization. As it has been reviewed previously, many short peptides generated from pork products have potential DPP-IV inhibitory activity, and thus, they may play a role in promoting satiety via avoiding GLP-I cleavage. Further studies are needed to investigate this promising hypothesis.

Peptones can stimulate CCK synthesis in enteroendocrine cell line STC-1, in rats and in humans, being later demonstrated that peptones activate CCK gene transcription in STC-1 enteroendocrine cells by a DNA sequence named peptone-response element housed in the CCK promoter [209,210]. In the same line, pork peptone treatment leads to a dose-dependent CCK secretion from STC-1 cells in higher amounts than soybean β-conglycinin and chicken peptones. Pork, chicken, and beef peptones have been shown to bind similarly to solubilized brush–border membrane of rat small intestine, with higher ligand affinity than other peptones from beef liver and egg white. Finally, it has been demonstrated that after orogastric administration of pork peptone preload to rats, a more marked food intake suppression than chicken peptone is produced. In fact, pork peptone preload showed a dose-dependent effect on food suppression intake, similarly to soybean β-conglycinin preload [205].

Besides their interaction with gut hormones synthesis and secretion, food-derived peptides could interact with the peripheral opioid receptors and indirectly induce gluconeogenesis that participates in the maintenance of satiety and reduction of food intake [203].

#### 8.6.3. Brain Health Promoting and Neuronal-Related Activities

The transportability of several di- and tripeptides through the blood–brain barrier has been evaluated via in vitro cell membrane-based experiments, ex vivo sliced brain tissues or ventricle plexus, and in vivo brain perfusion experiments. The criteria which makes a peptide transportable consists of a dipeptide skeleton and having a capability to bind the PHT1 peptide transporter [211,212]. Dipeptides such as GG, GL, GP, HL, MM, YP, and carnosine have been suggested to be transported by PHT1, being interesting to remark that the antioxidant dipeptide carnosine and the DPP-IV inhibitory dipeptide GP have been reported to be present in Spanish dry-cured ham [213].

In this context, the intake of soy peptide preparations, composed mainly of di- and tripeptides, was reported to modulate the levels of certain neuroactive amino acids in the adult mouse brain and increase serum dopamine level and improve cognitive dysfunction in subjects with mild cognitive impairment [214]. Furthermore, these peptide preparations have been proved to suppress cognitive decline by induction of neurotrophic factors in SAMP8 mice [215]. More precisely, dipeptides such as FL, GR, IY, LH, MKP, SY, WL, WM, WY, YL, YP, and YW were correlated to exert a functional activity in the brain, but further experiments are necessary to clarify their possibility to be assimilated (Matsui et al., 2020). Moreover, the peptide γ-EW and the W residue-rich whey protein hydrolysate have been described to increase serotonin synthesis and to have effects possibly related to serotonergic activation and anti-oxidative activity against anxious depression in C57BL/6 mice [216].

P-specific peptidases prolyl-endopeptidases (PEP) and DPP-IV belong to a group of serine proteases that play a role in the pathophysiological mechanisms of affective disorders. PEPs are involved in cleavage of hormones and small neuropeptides and are structurally and functionally closely related to those of the DPP-IV sub-families. Behavioral disorders are related to an increase in the activity of these enzymes in brain structures of rats [217]. P residue confers resistance to proteolysis, but serum DPP-IV can hydrolyze opioid peptides, reducing their half-life [218]. For this reason, the previously described DPP-IV inhibitory peptides may exert opioid effects by reducing the proteolysis of bioactive peptides, in addition to their potential due to their amino acid sequence for acting as opioid agonists.

In this regard, DPP-III shows a high affinity and ability to cleave endogenous opioid peptides and angiotensin II. Some short peptide inhibitors have been reported, for instance, YY, YF, LR, RR, denoting the relevance of basic or aromatic and hydrophobic residues in the sequences [219,220]. An in silico analysis predicted many DPP-III inhibitory sequences within meat proteins to be potentially released. Particularly, β-subunit of hemoglobin could present a high potential as source of such bioactive peptides [219]. Dry-cured ham is also a potential source of DPP-III inhibitors such as HK, HP, LR, and VY that are probably generated during processing [22].

## 9. Experimental Relationships between Sensory and Bioactive Peptides

The discovery of bioactive and sensory peptides naturally generated during processing can represent an important increase in the added value of dry-cured meats, as well as the possibility of optimizing the manufacturing process in order to favor the generation of compounds of interest. Based on their structure features, several bioactive peptides exert multifunctional properties due to interlinking metabolic pathways by controlling specific functions and to the fact that bioactive peptides might serve as signaling molecules [101]. According to scientific reports, peptides derived from foods possess dual properties; that is, they exhibit biological functions as well as affect the food taste [42]. For example, many bitter dipeptides show ACE inhibitory activity due to structural homologies [72].

In this review, some taste-active and potentially multifunctional di- and tripeptides derived from pork proteins have been reported, with an emphasis on dry-cured ham-derived peptides. Good examples are bitter peptides GP and PP, and sweet peptide AA, which may act as hypotensive and hypoglycemic agents [75,90,91]. EA and VE could exert the same bioactivities, but also have umami taste [86,186]. VY possesses hypotensive and hypoglycemic potentials but while the first could have anti-obesity effects, VY might be an antioxidant, and opioid compound, while having bitter taste [75,91,154,184]. LL has been described as Immunomodulator, hypoglycemic, antihypercholesterolemic, and bitter [91,142,143]. In addition, the umami peptides EL, EV, RL, EEL, and ESV, identified in Jinhua dry-cured ham, have been reported to exert moderate in vitro ACE-I inhibitory activity [14,86], while dipeptides DA, DD, EE, and ES exerted in vitro HMG-CoAR inhibition [86,91,101,146].

More evidence is needed in order to determine a possible link between the potential bioactivity and the sensory properties of di- and tripeptides, which will highlight the possibilities for application in the food industry. Di- and tripeptides with such multifunctional bivalence (taste-activity and bioactivity) create promising perspectives for processed foods with improved taste and increased functionality.

## 10. Conclusions

This review reports the current knowledge on those di- and tripeptides that are generated during the long processing of dry-cured ham. Some of these small peptides have shown significant bioactivity with positive effects on health and also sensory properties that contribute to taste. This is evidence that pork muscle proteins constitute a source of encrypted short health-promoting and taste-active peptides which can be released by proteolysis during processing and/or gastrointestinal digestion. Further, the amino acid composition and structure properties of such peptides are key factors determining their affinity for targeting molecules implicated in disease-related signalizations and taste perception. In general, the low toxicity, high specificity and higher probability of being absorbed intact, make di- and tripeptides present in dry-cured pork products of great interest and relevance for consumers’ health.

## Figures and Tables

**Table 1:** L-amino acid tastes listed by amino acid hydropathy.

Amino Acid	Abbreviation ^a^	Threshold (μM)	Taste
Isoleucine	I	7.51	Bitter
Valine	V	4.76	Sweet and bitter
Leucine	L	6.45	Bitter
Phenylalanine	F	6.61	Bitter
Cysteine	C	0.063	Sulfurous
Methionine	M	3.72	Sweet and bitter
Alanine	A	16.2	Sweet
Glycine	G	30.9	Sweet
Threonine	T	25.7	Sweet and bitter
Serine	S	0.029	Sweet
Tryptophan	W	2.29	Bitter
Tyrosine	Y	ND	Bitter
Proline	P	15	Sweet and bitter
Histidine	H	1.23	Bitter
Glutamate	E	0.063	Umami, bitter, salty, sour
Glutamic acid	Q	9.77	Sweet
Aspartic acid	D	0.182	Umami, bitter, salty, sour
Asparagine	N	1.62	Bitter
Lysine	K	0.708	Salty, sweet, bitter
Arginine	R	1.20	Sweet and bitter

^a^ Amino acids are given in one-letter code. Data shown in this table obtained from [69,71,72].

**Table 2:** Taste characteristics of dry-cured ham (DCH)-derived di- and tripeptides.

Sequence ^a^	Origin (Curing Months)	Reference
***Bitter ^b^***		
IV, LE, ID, and PL	Serrano DCH (8 m)	[40]
GF and LL	Parma DCH (12 m)	[81]
AD, DL, EA, EE, EF, EI, EL, GP, IF, IL, KP, LA, LG, LL, PA, PK, PL, PP, RG, VE, VF, VY	In silico	[75]
PG and VG	Norwegian DCH (21 m)	[82]
PL	Jinhua (DCH) (8 m)	[83]
GL	Prosciutto-like DCH (22 m)	[32]
PA and VG	Traditional and low-salted Spanish DCH (6, 12, 18, and 24 m)	[84,85]
***Umami***		
DE, EA, EE, EK, EL, VE	In silico	[75,86]
EE, EF, EK, and DA	Jinhua DCH (6 m)	[73]
EL, EV, RL, EEL, and ESV	Jinhua DCH (6 m)	[15]
DA, DG, EE, ES, EV, and VG	Traditional and low-salted Spanish DCH (6, 12, 18, and 24 m)	[84,85]
AH	Spanish DCH (18 m) Jinhua (6 m)	[83,87]
EE	Prosciutto-like DCH (22 m)	[32]
DK	Spanish DCH (9 m)	[22]
***Sweet***		
AA, AAA, AGA, AGG, EV, GAG, and GGA	In silico	[75]
AA and GAG	Spanish DCH (2 m)	[88,89]
AA	Traditional and low-salted Spanish DCH (6, 12, 18, and 24 m) Prosciutto-like DCH (22 m)	[32,90]
***Sour***		
AED, DD, DE, DV, DEE, DES, ED, EV, VD, and VE	In silico	[75]
VE, GE, and DV	Serrano DCH (8 m)	[40]
DE	Spanish DCH (12 m)	[88,91]
***Salty***		
DE	Spanish DCH (12 m)	[22,88]
AR	Jinhua (6 m)	[83]
***Kokumi***		
γ-EI, γ-EL, γ-EF and γ-EY	Parma DCH (18, and 24 m)	[81,92]
γ-EF, γ-EW and γ-EY	Prosciutto DCH (14, 21, and 34 m)	[93]

^a^ Peptides are given in one-letter code. DCH indicated dry-cured ham^. b^ Basic tastes given in italics.

## Data Availability

Data will be made available on request.
